# Mutagenicity and Acute Oral Toxicity Test for Herbal Poultry Feed Supplements

**DOI:** 10.1155/2018/9412167

**Published:** 2018-05-10

**Authors:** Boddapati Srinivasa Rao, C. V. Chandrasekaran, H. S. Srikanth, Murugan Sasikumar, R. Edwin Jothie, Begum Haseena, Bethapudi Bharathi, Ramasamy Selvam, D'Souza Prashanth

**Affiliations:** R&D, Natural Remedies Pvt. Ltd., Plot No. 5B, Veerasandra Industrial Area, 19th KM Stone, Hosur Road, Bangalore, Karnataka 560100, India

## Abstract

Herbal products are being used and trusted globally for thousands of years for their health benefits and limited side effects. Globally, a general belief amongst the consumers is that herbal supplements are always safe because they are “natural.” But later, research reveals that they may not be safe. This raises concern on their safety and implications for their use as feed supplement or medicine. Toxicity testing can reveal some of the risks that may be associated with use of herbs, therefore avoiding potential harmful effects. The present study was designed to investigate five poultry feed supplements (PFS), EGMAX® (to revitalize ovarian activity), FEED-X**™** (feed efficiency enhancer), KOLIN PLUS**™** (natural replacer of synthetic choline chloride), PHYTOCEE® (natural defence enhancer), and STODI® (to prevent and control loose droppings), for their possible mutagenicity and toxicity. Bacterial reverse mutation (BRMT) and acute oral toxicity tests were employed to assess the PFS for their possible mutagenicity and toxicity. Results indicated that the PFS were devoid of mutagenic effects in BRMT and showed higher safety profile in rodent acute oral toxicity test.

## 1. Introduction

Poultry industry is one of the most fertile areas to ease out the pressure of population on crop cultivation. Amongst the livestock based vocations, poultry farming occupies a pivotal position due to its enormous potential to bring about rapid economic growth with low input investment [1, 2]. Though the industry continues to face numerous challenges on global basis, disease outbreaks and implementation of biosecurity programs are the top challenges to increase the productivity of poultry industry [3].

Products that are effective, environmentally, and user friendly will provide safe, economical, and long-term success to poultry health programs [4]. In this context, EGMAX, FEED-X, KOLIN PLUS, PHYTOCEE, and STODI were developed as PFS by Natural Remedies Pvt. Ltd. and adequate studies have been performed to prove efficacy of these feed supplements.

EGMAX minimizes the gap between standard and actual egg production. It minimizes hairline cracks of eggs and also improves fertility in female breeders. FEED-X optimizes the feed efficiency in poultry. It improves the gastrointestinal function for better absorption of nutrients and also improves carcass characteristics. KOLIN PLUS is a natural replacer of synthetic choline chloride. It reduces the abdominal and liver fat levels. It maintains the healthy liver and supports the growth of poultry birds. PHYTOCEE enhances and maintains the immune competence. It protects the bird from the ill effects of production stress and other stresses. It compensates the stress-induced depletion of Vitamin* C. STODI* prevents and controls wet litter in poultry during high risk periods, for example, disease outbreak, susceptible season or age, changes in environmental conditions, peak production/growth, and dietary errors. It helps to prevent/treat the noninfectious diarrhoea. It is also supportive to treat infectious diarrhoea along with antibiotic treatment.

Even though proofs of efficacy on these PFS are available [5, 6], it is vital to assess safety to support their long-term use. Hence, the current study evaluated the PFS for their possible toxic potential by using* in vitro* BRMT and* in vivo* acute oral toxicity test.

In the current study, BRMT was performed using AMES MPF fluctuation procedure in microplate format (MPF). The AMES MPF test is a fluctuation method which is different from traditional AMES preincubation and plate incorporation method. AMES MPF uses liquid media and 384-well microplates with readout based on a colour change [7]. Several researchers stipulated that the AMES MPF assay is a consistent prophetic gadget that can be used like the usual AMES test to evaluate compounds for mutagenicity [8]. The main advantage of this test is that it can be used with much less test chemical than the conventional AMES methods. AMES MPF requires less hands-on time, S9, and plastic ware and can be automated [9].

Acute oral toxicity test is usually an initial screening step in the assessment and evaluation of the toxic characteristics of test substances in* in vivo*. Acute toxicity is involved in estimation of LD_50_ (the dose which has proved to be lethal (causing death) to 50% of the animals tested) and also in examining the adverse effects that occurs on first exposure to single oral dose administration of test substance [10, 11].

## 2. Material and Methods

### 2.1. Test Substances

EGMAX, FEED-X, KOLIN PLUS, PHYTOCEE, and STODI are herbal PFS developed and manufactured by Natural Remedies Pvt. Ltd., Bangalore, India.

EGMAX, FEED-X, KOLIN PLUS, PHYTOCEE, and STODI are standardized botanical powder found to contain not less than 0.1% aloin A and aloin B, 0.5% andrographolide, 8.0% polyphenols, 0.5% of gallic acid, and 0.20% punicalagin, respectively.

### 2.2. Chemicals

2-Aminoanthracene, 2-nitrofluorene, N^4^-aminocytidine, 9-aminoacridine, and 4-nitroquinoline-n-oxide were procured from Sigma-Aldrich. Dimethyl sulphoxide (DMSO) and carboxymethylcellulose sodium salt (CMC) were procured from Himedia Laboratories Pvt. Ltd.

**Figure 1:**
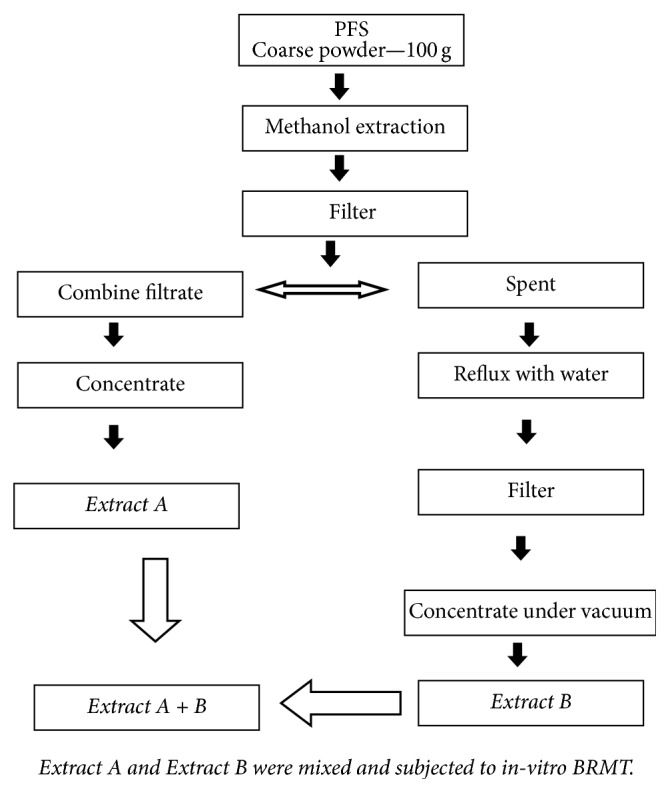
Extraction procedure of PFS.

PFS were individually extracted as per Figure 1. Resulting extracts (extract A + B) were subjected to* in vitro* BRMT. PFS as such were subjected for* in vivo* acute oral toxicity evaluation.

AMES MPF™ Penta I test kit was procured from Xenometrix. The kit contains* Salmonella typhimurium (S. typhimurium)* (TA 98, TA 100, TA 1535, and TA 1537) and* Escherichia coli (E. coli) (WP2 uvrA)* strains, Aroclor 1254 induced rat liver microsomal fraction (S9), buffer salts, G-6-P, NADP, growth media, exposure media, and reversion indicator media. Genotyping of tester strains was performed by Xenometrix and verified with the help of certificate of analysis. Tester strains were analyzed for their spontaneous mutation rate and genotypic characteristics such as mutations at* his*,* bio* loci (histidine and biotin dependency),* rfa* mutation (defective lipopolysaccharide (LPS) layer that coats the cell surface), and* uvrB* deletion (eliminating the accurate excision repair mechanism) and for the presence of plasmids pKM101 (enhancing error-prone recombinational DNA repair pathway and also conferring ampicillin resistance) by Xenometrix and confirmed their genetic integrity.

### 2.3. Animals

Female albino Wistar rats that were bred and reared at Natural Remedies Pvt. Ltd., Bangalore, India, were used in acute oral toxicity studies.

### 2.4. *In Vitro* BRMT

BRMT was used to identify the ability of test substance to induce reverse mutation at histidine loci in* S. typhimurium* strains like TA 98, TA 100, TA 1535, and TA 1537 and tryptophan loci in* E. coli (WP2 uvrA)*. BRMT with fluctuation method (AMES MPF Penta I-Micro Plate Format mutagenicity assay) was followed to determine the possible genotoxic potential of the four PFS. Based on solubility and precipitation test, DMSO (4%) was used as vehicle control. Cytotoxicity test was conducted using TA 98 strain in both absence and presence of S9 (4.5%) to select the test concentrations for mutagenicity test. For cytotoxicity test, percentage of reduction was determined and the concentrations which showed cell reduction >50% were expelled for mutagenicity testing [12]. In the mutagenicity test,* S. typhimurium* and* E. coli strains *were treated with PFS/vehicle control/appropriate positive controls in absence and presence of S9 (4.5%) in triplicate along with indicator media and kept for 48 hours of incubation at 37 ± 2°C. Revertant colonies were counted and positive response was determined [13, 14].

### 2.5. *In Vivo* Acute Oral Toxicity Test

The animal experiment was conducted according to the CPCSEA (Committee for the Purpose of Control and Supervision of Experiments on Animals) guidelines and after approval by the Institutional Animal Ethics Committee (IAEC). Acute oral toxicity study was performed as per the OECD test guideline for testing of chemicals (Test Number 420, Section 4: Health Effects) acute oral toxicity, fixed dose procedure adopted on 17 December 2001 [10]. Healthy female albino Wistar rats (8–12 weeks) were accommodated in polypropylene cages and temperature was maintained at 23 ± 2°C with 12 hours of dark and light cycle each. The rats were fed with standard laboratory pelleted feed. The rats were fasted overnight before and 3 hours after the administration of test substances. Test substances were suspended in 0.5% CMC and were administered by oral route to rats at the limit dose of 5 g/kg body weight in a sequential manner. On the day of dosing, all the animals were observed for mortality and clinical signs for first 10 minutes, 30 minutes, 1 hour, 2 hours, 4 hours, and 6 hours after dosing and thereafter twice daily for mortality and once a day for clinical signs, for 14 days. Daily cage side observations included changes in the skin and fur, eyes and mucous membrane, also respiratory, circulatory, autonomy, and central nervous systems, and somatomotor activity and behavioral pattern. Particular attention was directed to the observation of tremors, convulsions, salivation, diarrhoea, lethargy, sleep, and coma. Individual animal bodyweight was recorded shortly before the test substances administration and weekly thereafter; changes in body weight gain were also calculated. After the observation period of 14 days, all surviving rats were euthanized and subjected to complete necropsy.

### 2.6. Statistical Analysis

Data is represented as mean ± standard deviation (SD) of three replicates. Statistical significance was evaluated using one-way analysis of variance (ANOVA) followed by Dunnett's multiple comparisons using GraphPad Prism 5.0 (GraphPad Software Inc., San Diego, CA). Statistical significance level was set at *P* < 0.05.

## 3. Results

### 3.1. Cytotoxicity

Cytotoxicity test on PFS was conducted by using TA 98 strain in both presence and absence of S9 to select appropriate concentrations for the main study.  Table 1 shows the cytotoxicity experiment results after treatment with PFS.

Cytotoxicity is determined by calculating the percentage of reduction on cell viability with the following formula:

(1) 100−Mean  OD  value  of  treated  controlMean  OD  value  of  untreated  control×100.

As per OCED TG 471 percentage of reduction on cell viability up to 50% is admissible. Concentrations of test substances which showed reduction up to 50% were selected as highest dose for mutagenicity test. Thus the concentration which elicited reduction in cell viability more than 50% was eliminated for mutagenicity test.

Based on the cytotoxicity results, the concentrations of test substances which showed less than 50% reduction in cell viability (Table 2) were conducted for mutagenicity study.

### 3.2. Mutagenicity Test

PFS were assessed for their genotoxicity potential by using BRMT-fluctuation assay and found to be negative, that is, not mutagenic to the tester strains at all tested concentrations in both presence and absence of metabolic activation. In vehicle and untreated controls, revertant colonies were developed in normal range. In the PFS treated concentrations, there was no statistically significant difference and there was no two fold increase in revertant colonies over the vehicle control values (Tables 3–6). Positive controls showed statistical significance response (*P* < 0.05) and also showed fold increase more than two with respect to the vehicle control and thus demonstrated the validity of test (Tables 3–6).

### 3.3. Acute Oral Toxicity Test

PFS were evaluated for their acute oral toxicity by administering them as a single oral dose to female albino Wistar rats. PFS were administered orally in a sequential manner to five rats at the limit dose level of 5000 mg/kg bodyweight. On the day of treatment, animals were observed for mortality and clinical signs for first 10 minutes, 30 minutes, 1 hour, 2 hours, 4 hours, and 6 hours after dosing and thereafter twice a day for mortality and once a day for clinical signs for 14 days. The bodyweight of rats was recorded and weekly bodyweight gain was calculated. After the 14 days of observation period, all surviving rats were euthanized and subjected to complete necropsy.

The PFS treated rats survived till the end of the study period and did not show any treatment related adverse clinical signs immediately following dosing and during the 14 days' observation period. In sighting and main studies, treatment with PFS did not reveal any major adverse effects on the body weight gain during the 14 days' observation period. Overall, the percent bodyweight gain during the 14 days' observation period was found to be normal in all the PFS treated rats. On necropsy, no major gross pathological changes were observed in any of the PFS treated rats (Tables 7 and 8). Based on the findings of the present study, all PFS were found to be safe after oral administration as a single dose of 5000 mg/Kg bodyweight to female albino Wistar rats.

## 4. Discussion

The innovation, development, and marketing of herbal feed supplements are currently the growing segments of the feed industry. Functional feeds may be recognized as feeds or feed ingredients that have supplementary health or physiological benefits apart from their regular nutritional value. This inclination is ambitious by multiple factors, chiefly due to the current consumer perceptions: the primary and leading one being “natural is good” and other minor ones, such as the growing cost of pharmaceuticals and their adverse ancillary effects, the persistent marketing campaign, and the increasing perception of the need of a healthy feed and its significance in the health and homeostasis organism conditions [15].

Though the vital fact is that herbal feed supplements, including the entry of new functional feed ingredients, are imperative for their acceptance as the novel and modern forms to benefit of natural substances, due to the rapid expansion in this area, the development of several aspects is considered as it could influence the future of the market. The functional properties of many herbal preparations, in particular, are being investigated for potential use as novel feed supplements in animal healthcare especially in poultry farming [15]. Even though the availability of scientific evidence is rapidly enlightening, the crucial aspect concerns the validation of their safety. Herbal preparations are generally assumed to be safe [16, 17] and certified as Generally Recognized as Safe (GRAS) by the Food and Drug Administration (FDA). But due to the complex phytochemical nature, undesired residual presence, and transformation of bioactive ingredients by the metabolites in tissues, the safety of the same is dubious. The first step to fulfill the safety concerns is by employing toxicological tests to validate their safety. In this context PFS, EGMAX, FEED-X, KOLIN PLUS, PHYTOCEE, and STODI were subjected to toxicological screening using BRMT and acute oral toxicity studies.

The BRMT and acute oral toxicity test are recommended by several regulatory agencies (Organisation for Economic Cooperation and Development, Food and Drug Administration, and International Conference on Harmonisation) for substance evaluation to determine the safety. Due to its simplicity and relatively low cost, BRMT is commonly employed as an initial screening method for genotoxic activity [18, 19]. EGMAX, FEED-X, KOLIN PLUS, and STODI at the tested concentrations did not induce any significant mutagenicity in all the tester strains of* S. typhimurium* (TA98, TA100, TA1535, and TA1537) and* E. coli (WP2 uvrA)* both with and without metabolic activation. This indicates that our formulations cause neither any frame shift mutations in the tester stains TA98 and TA1537 nor base pair mutations in the tester strains TA100, TA1535, and* E. coli WP2 uvrA*.

EGMAX contains* Aloe barbadensis* (*A. barbadensis*) and* Solanum xanthocarpum (S. xanthocarpum)*, found to be nonmutagenic in both presence and absence of metabolic activation in BRMT. Similarly, water and methanolic extract of* A. barbadensis* were reported to be nonmutagenic to* S. typhimurium* strains TA 98 and TA 100 [20]. Aloin, phytochemical marker of EGMAX, was reported to be nonmutagenic in AMES test at concentrations ranging within 50–250 *μ*g/plate [21]. EGMAX was well tolerated by albino Wistar rats and LD_50_ was found to be more than 5000 mg/kg. Similar to the above finding,* Solanum xanthocarpum* was reported to be safe with an LD_50_ value of >2000 mg/kg [22].

FEED-X composed of* Andrographis paniculata (A. paniculata), P. granatum*, and* E. officinalis* was found to be nonmutagenic. Several reports demonstrated that extracts of* A. paniculata, P. granatum*, and* E. officinalis* were found to be safe in* in vitro*,* in vivo*, and clinical trials.* A. paniculata* standardized to andrographolide was reported to be nongenotoxic in AMES, chromosomal aberration, and micronucleus tests [13]. Aqueous and lipophilic pomegranate peel extracts have been reported to possess antimutagenic property [23]. Aqueous pomegranate fruit extracts were observed to be nontoxic to mice and able to protect against cyclophosphamide-induced oxidative DNA damage [24, 25]. LD_50_ of* A. paniculata* extract was found to be greater than 5 g/kg in rodents at the single oral administration [13]. Also* A. paniculata* was reported to be devoid of reproductive toxicity [26, 27]. In the current acute oral toxicity study, treatment with FEED-X did not produce any treatment related adverse effect up to the dose level of 5000 mg/kg in rats. Based on the reported evidence on the ingredients of FEED-X and of the current study, it is imperative to state that FEED-X is devoid of mutagenicity and toxicity.

KOLIN PLUS, an herbal amalgamation of* Acacia nilotica (A. nilotica)* and* Curcuma longa (C. longa)*, was found to be nonmutagenic. Similarly, acetone extract of* A. nilotica* was reported to exhibit antimutagenic activity against 2-amino fluorine [28].* C. longa* was reported to be nongenotoxic to rodents [29]. Polysaccharide extract of* C. longa* was found to be nonmutagenic to* S. typhimurium* strains like TA98 and TAMix. Also it was found to be safe up to 5000 mg/kg to rodents after a single oral administration [7]. LD_50_ of* Acacia arabica* was found to be greater than 2000 mg/kg [30]. Safety review on the ingredients and current study signified that KOLIN PLUS can be considered as nonmutagenic and safe.

PHYTOCEE used in poultry feeds was reported to be nonmutagenic to TA98, and TAMix strains in both the absence and presence of metabolic activation [6]. The review on the herbal constituents revealed that* E. officinalis*,* Ocimum sanctum (O. sanctum)*, and* Withania somnifera (W. somnifera)* were found to be safe. Antigenotoxic activity of* W. somnifera* against malathion-induced DNA damage in mice leucocytes was reported [31].* W. somnifera* extract was reported to protect the albino mice against toxicity induced by lead nitrate [32].* In vivo* study on* W. somnifera* root extract in mice reported nongenotoxic effect as evident from no significant changes of chromosome morphology [33]. Administration of ethanolic extract of* O. sanctum* at a dose 5 g/kg body weight did not cause micronucleus induction in bone marrow cells of rats. Ethanol extract of* O. sanctum* was reported to be antimutagenic to cyclophosphamide-induced micronucleus formation at a dose of 5 g/kg body weight. The antimutagenicity of* O. sanctum* was attributed to the enhanced levels of liver detoxification enzymes.* O. sanctum* has been reported to have anticlastogenic effect against Mitomycin C- and chromium-induced genotoxicity in human peripheral blood lymphocytes [34]. In an* in vivo* chromosomal aberration study, leaves of* O. sanctum* exhibited protective effect on chromium- and mercury-induced structural chromosomal aberrations [35]. In this study, PHYTOCEE is safe up to 5000 mg/kg after oral administration to rats. Overall, PHYTOCEE ingredients and as a formulation are found to be nonmutagenic and safe for oral consumption.

STODI is a herbal blend of* P. granatum*,* A. arabica*,* H. antidysenterica*,* A. paniculata*, and* Terminalia bellerica (T. bellerica)*. In this study, STODI was found to be nonmutagenic to* S. typhimurium* and* E. coli* strains in presence and absence of metabolic activation. The antimutagenic effect of* H.antidysenterica* was demonstrated against NaN_3_(sodium azide) and MMS (methyl methane sulfonate) in TA 97a, TA 100, TA 104, and TA 102 tester strains [36]. The current study revealed the LD_50_ of STODI more than 5000 mg/kg body weight in rats. Aqueous, ethanol, and hydroalcoholic extracts of* H. antidysenterica* seeds are reported to be safe up to 2000 mg/kg body weight in rats [37, 38]. Ethanol extract of* H. antidysenterica* leaves was reported to be safe up to 2000 mg/kg after single oral dose in rats [39]. Acetone extract of* T. bellerica* was reported to be antimutagenic against 4-O-nitrophenylenediamine (NPD) and NaN_3_ in AMES* Salmonella*/microsome assay [40].* T. bellerica* was reported to possess LD_50_ value higher than 2000 mg/kg body weight of Wistar rats [41]. Based on the above reports and the current study, it is prudent to state that STODI is safe for oral consumption.

The current study reckoned that PFS, EGMAX, FEED-X, KOLIN PLUS, PHYTOCEE, and STODI were nonmutagenic in* S. typhimurium* and* E. coli* strains in presence and absence of metabolic activation. In acute oral toxicity studies, no major gross pathological changes were observed in any of the PFS treated animals. PFS fed rats exhibited comparable weight gain. This suggested that the PFS did not exert any deleterious effects on the general health status and metabolic growth of the rats. Under our test conditions, the oral administration of EGMAX, FEED-X, KOLIN PLUS, PHYTOCEE, and STODI was found to be safe up to 5000 mg/kg in female albino Wistar rats. Based on the results, all the PFS were declared as unclassified in the hazard category according to Globally Harmonized System.

In conclusion, the results of this investigation revealed that the four poultry feed supplements EGMAX, FEED-X, KOLIN PLUS, and STODI were nonmutagenic in* in vitro* BRMT. Also it was concluded that the five poultry feed supplements EGMAX, FEED-X, KOLIN PLUS, PHYTOCEE, and STODI were nontoxic to rats and LD_50_ was found to be more than 5000 mg/kg rat body weight.

## Figures and Tables

**Table 1:** Cytotoxicity test results.

EGMAX				FEED-X				Kolin PLUS				STODI			
Treatment (*μ*g/ml)	S9	Average OD (*n* = 3)	% of reduction	Treatment (*μ*g/ml)	S9	Average OD (*n* = 3)	% of reduction	Treatment (*μ*g/ml)	S9	Average OD (*n* = 3)	% of reduction	Treatment (*μ*g/ml)	S9	Average OD (*n* = 3)	% of reduction
NC	−S9	0.335	NA	NC	−S9	0.391	NA	NC	−S9	0.396	NA	NC	−S9	0.409	NA
	+S9	0.475	NA		+S9	0.455	NA		+S9	0.417	NA		+S9	0.428	NA
VC	−S9	0.259	NA	VC	−S9	0.317	NA	VC	−S9	0.318	NA	VC	−S9	0.325	NA
	+S9	0.361	NA		+S9	0.385	NA		+S9	0.387	NA		+S9	0.326	NA
78.1	−S9	0.229	12	39.2	−S9	0.308	3	19.1	−S9	0.322	0^∧^	39.2	−S9	0.322	1
	+S9	0.332	8		+S9	0.355	8		+S9	0.364	6		+S9	0.332	0^∧^
156.2	−S9	0.187	28	78.1	−S9	0.326	0^∧^	39.0	−S9	0.235	26	78.1	−S9	0.330	0^∧^
	+S9	0.333	8		+S9	0.403	0^∧^		+S9	0.425	0^∧^		+S9	0.377	0^∧^
312.5	−S9	0.132	49	156.2	−S9	0.353	0^∧^	78.1	−S9	0.191	40	156.2	−S9	0.338	0^∧^
	+S9	0.315	13		+S9	0.407	0^∧^		+S9	0.408	0^∧^		+S9	0.347	0^∧^
625	−S9	0.111	57^#^	312.5	−S9	0.332	0^∧^	156.2	−S9	0.336	0^∧^	312.5	−S9	0.273	16
	+S9	0.236	35		+S9	0.378	2		+S9	0.413	0^∧^		+S9	0.342	0^∧^
1250	−S9	0.113	57^#^	625	−S9	0.302	5	312.5	−S9	0.372	0^∧^	625	−S9	0.223	31
	+S9	0.165	54^#^		+S9	0.330	14		+S9	0.414	0^∧^		+S9	0.319	2
2500	−S9	0.100	61^#^	1250	−S9	0.252	20	625	−S9	0.364	0^∧^	1250	−S9	0.167	49
	+S9	0.167	54^#^		+S9	0.276	28		+S9	0.306	21		+S9	0.317	3

^#^Cytotoxic concentration which showed >50% reduction in cell viability over the control. ^∧^No reduction on cell viability over control. NA: not applicable; NC: negative control; VC: vehicle control (DMSO-4%).

**Table 2:** Concentrations of test substances selected for main mutagenicity test.

Test substance	S9	Test substance concentrations (*μ*g/ml)
EGMAX	−S9	19.5 to 312.5
	+S9	39.0 to 625
FEED-X	−S9	78.1 to 1250
	+S9	78.1 to 1250
KOLIN PLUS	−S9	39.0 to 625
	+S9	39.0 to 625
STODI	−S9	78.1 to 1250
	+S9	78.1 to 1250

**Table 3:** EGMAX, mutagenicity test results.

Strain	S9	Untreated control	Vehicle control (DMSO)	EGMAX						Positive control
				19.5 *μ*g/ml	39.0 *μ*g/ml	78.1 *μ*g/ml	156.2 *μ*g/ml	312.5 *μ*g/ml	625 *μ*g/ml	
*S. typhimurium*										
TA 98	−S9	1.00 ± 1.73	0.67 ± 1.15	0.33 ± 0.58	1.67 ± 0.58	0.67 ± 0.58	0.33 ± 0.58	0.67 ± 1.15	NA	46.00^*∗*^ ± 1.00
	+S9	1.67 ± 1.53	1.33 ± 0.58	NA	3.67 ± 0.58	0.67 ± 1.15	1.00 ± 1.00	0.67 ± 0.58	2.33 ± 0.58	45.33^*∗*^ ± 1.53
TA 100	−S9	1.67 ± 0.58	2.00 ± 1.00	1.33 ± 1.53	1.00 ± 0.00	0.00 ± 0.00	1.33 ± 0.58	1.33 ± 1.15	NA	43.33^*∗*^ ± 0.58
	+S9	5.67 ± 3.06	3.00 ± 0.00	NA	3.33 ± 0.58	3.33 ± 0.58	4.67 ± 0.58	4.33 ± 2.08	3.00 ± 1.00	48.00^*∗*^ ± 0.00
TA 1535	−S9	1.33 ± 1.15	0.00 ± 0.00	1.67 ± 2.08	1.33 ± 1.53	0.00 ± 0.00	0.67 ± 1.15	0.33 ± 0.58	NA	48.00^*∗*^ ± 0.00
	+S9	0.00 ± 0.00	0.67 ± 0.58	NA	0.67 ± 1.15	0.67 ± 0.58	0.33 ± 0.58	1.33 ± 1.53	1.33 ± 1.53	43.33^*∗*^ ± 4.04
TA 1537	−S9	1.00 ± 0.00	1.00 ± 1.00	0.67 ± 0.58	0.67 ± 0.58	0.00 ± 0.00	0.33 ± 0.58	1.33 ± 0.58	NA	40.67^*∗*^ ± 0.58
	+S9	0.33 ± 0.58	2.00 ± 2.00	NA	0.33 ± 0.58	1.33 ± 0.58	1.00 ± 1.00	0.67 ± 1.15	1.33 ± 1.53	37.67^*∗*^ ± 1.15
*E. coli*										
*WP2 uvrA*	−S9	1.00 ± 1.00	0.00 ± 0.00	0.33 ± 0.58	0.33 ± 0.58	0.00 ± 0.00	1.00 ± 0.00	0.67 ± 1.15	NA	41.33^*∗*^ ± 6.43
	+S9	1.00 ± 1.00	0.67 ± 0.58	NA	0.67 ± 0.58	1.00 ± 0.00	1.00 ± 0.00	1.33 ± 0.58	0.67 ± 0.58	37.33^*∗*^ ± 2.52

Revertant colonies *n* = 3 (mean ± SD). ^*∗*^Statistically significant (*P* < 0.05); NA: not applicable; positive controls. With S9: 2-aminoanthracene (i) 5 *μ*g/ml, TA 98, TA 100, TA 1535, and TA 1537; (ii) 50 *μ*g/ml, *E. coli WP2 uvrA*; without S9: 2-nitrofluorene—2 *μ*g/ml, TA 98; 4-nitroquinoline-N-oxide—0.1 *μ*g/ml, TA 100; N^4^-aminocytidine—100 *μ*g/ml, TA 1535; 9-amino-1,2,3,4-tetra hydro acridine hydrochloride—15 *μ*g/ml, TA 1537; 4-nitroquinoline-N-oxide—1.0 *μ*g/ml, *E. coli WP2 uvrA*.

**Table 4:** FEED-X, mutagenicity test results.

Strain	S9	Untreated control	Vehicle control (DMSO)	FEED-X					Positive control
				78.1 *μ*g/ml	156.2 *μ*g/ml	312.5 *μ*g/ml	625 *μ*g/ml	1250 *μ*g/ml	
*S. typhimurium*									
TA 98	−S9	0.67 ± 1.15	0.33 ± 0.58	2.00 ± 2.00	0.67 ± 1.15	1.00 ± 0.00	0.00 ± 0.00	2.33 ± 0.58	48.00^*∗*^ ± 0.00
	+S9	1.67 ± 1.15	1.33 ± 0.58	0.33 ± 0.58	1.67 ± 2.08	3.67 ± 1.53	1.33 ± 1.53	4.00 ± 1.00	34.67^*∗*^ ± 3.21
TA 100	−S9	1.67 ± 1.53	0.67 ± 0.58	0.67 ± 0.58	1.33 ± 0.58	1.67 ± 2.08	1.67 ± 1.15	2.33 ± 2.31	43.00^*∗*^ ± 2.00
	+S9	3.33 ± 2.08	7.33 ± 1.53	3.67 ± 1.53	6.33 ± 1.15	3.67 ± 2.08	3.33 ± 0.58	3.67 ± 1.53	48.00^*∗*^ ± 0.00
TA 1535	−S9	1.00 ± 0.00	0.67 ± 0.58	0.33 ± 0.58	1.00 ± 1.00	1.33 ± 0.58	0.67 ± 1.15	0.33 ± 0.58	48.00^*∗*^ ± 0.00
	+S9	0.00 ± 0.00	1.00 ± 0.00	0.67 ± 1.15	1.67 ± 1.53	1.33 ± 0.58	0.67 ± 1.15	0.00 ± 0.00	42.67^*∗*^ ± 1.15
TA 1537	−S9	0.00 ± 0.00	0.33 ± 0.58	0.00 ± 0.00	0.33 ± 0.58	1.67 ± 2.89	0.67 ± 0.58	1.00 ± 1.00	40.33^*∗*^ ± 1.53
	+S9	1.00 ± 1.00	1.00 ± 1.00	0.67 ± 0.58	0.67 ± 0.58	0.33 ± 0.58	0.67 ± 0.58	1.33 ± 1.53	42.00^*∗*^ ± 2.65
*E. coli*									
*WP2 uvrA*	−S9	0.33 ± 0.58	0.33 ± 0.58	0.00 ± 0.00	0.00 ± 0.00	0.33± 0.58	1.00 ± 1.00	0.67 ± 0.58	33.33^*∗*^ ± 3.21
	+S9	0.67 ± 0.58	1.33 ± 1.53	1.33 ± 1.53	1.33 ± 0.58	1.00 ± 1.73	0.33 ± 0.58	1.00 ± 0.00	39.00^*∗*^ ± 2.65

Revertant colonies *n* = 3 (mean ± SD). ^*∗*^Statistically significant (*P* < 0.05); positive controls. With S9: 2-aminoanthracene (i) 5 *μ*g/ml, TA 98, TA 100, TA 1535, and TA 1537; (ii) 50 *μ*g/ml, *E. coli WP2 uvrA*; without S9: 2-nitrofluorene—2 *μ*g/ml, TA 98; 4-nitroquinoline-N-oxide—0.1 *μ*g/ml, TA 100; N^4^-aminocytidine—100 *μ*g/ml, TA 1535; 9-amino-1,2,3,4-tetra hydro acridine hydrochloride—15 *μ*g/ml, TA 1537; 4-nitroquinoline-N-oxide—1.0 *μ*g/ml, *E. coli WP2 uvrA*.

**Table 5:** KOLIN PLUS, mutagenicity test results.

Strain	S9	Untreated control	Vehicle control (DMSO)	KOLIN PLUS					Positive control
				39.0 *μ*g/ml	78.1 *μ*g/ml	156.2 *μ*g/ml	312.5 *μ*g/ml	625 *μ*g/ml	
*S. typhimurium*									
TA 98	−S9	0.67 ± 1.15	2.00 ± 0.00	1.67 ± 1.15	0.33 ± 0.58	0.00 ± 0.00	0.00 ± 0.00	1.33 ± 0.58	48.00^*∗*^ ± 0.00
	+S9	0.67 ± 1.15	1.00 ± 1.00	1.33 ± 0.58	1.33 ± 0.58	3.00 ± 1.73	2.67 ± 2.51	0.67 ± 1.15	35.67^*∗*^ ± 2.52
TA 100	−S9	2.00 ± 2.65	1.67 ± 0.58	2.33 ± 0.58	2.00 ± 2.65	1.00 ± 1.73	1.33 ± 1.15	1.67 ± 2.08	45.33^*∗*^ ± 1.53
	+S9	5.33 ± 1.15	3.33 ± 0.58	4.00 ± 1.00	3.33 ± 1.53	2.33 ± 0.58	3.33 ± 1.53	2.00 ± 1.73	48.00^*∗*^ ± 0.00
TA 1535	−S9	2.00 ± 1.00	0.67 ± 1.15	0.67 ± 0.58	1.33 ± 2.31	0.67 ± 0.58	1.00 ± 1.00	1.33 ± 0.58	48.00^*∗*^ ± 0.00
	+S9	0.67 ± 0.58	0.33 ± 0.58	1.00 ± 0.00	3.00 ± 4.36	1.33 ± 1.53	2.00 ± 2.00	2.33 ± 2.52	42.67^*∗*^ ± 2.08
TA 1537	−S9	0.00 ± 0.00	0.00 ± 0.00	1.33 ± 0.58	0.33 ± 0.58	0.00 ± 0.00	0.67 ± 0.58	0.67 ± 0.58	40.67^*∗*^ ± 0.58
	+S9	1.00 ± 1.00	0.33 ± 0.58	1.00 ± 1.00	0.33 ± 0.58	0.33 ± 0.58	1.67 ± 1.53	0.67 ± 0.58	41.67^*∗*^ ± 2.08
*E. coli*									
*WP2 uvrA*	−S9	0.67 ± 0.58	1.00 ± 1.00	1.33 ± 0.58	1.00 ± 1.00	0.00 ± 0.00	0.00 ± 0.00	0.00 ± 0.00	43.00^*∗*^ ± 3.61
	+S9	0.67 ± 0.58	1.00 ± 1.00	1.67 ± 1.53	0.33 ± 0.58	0.33 ± 0.58	1.00 ± 1.00	0.67 ± 0.58	46.00^*∗*^ ± 2.65

Revertant colonies *n* = 3 (mean ± SD). ^*∗*^Statistically significant (*P* < 0.05); positive controls. With S9: 2-aminoanthracene (i) 5 *μ*g/ml, TA 98, TA 100, TA 1535, and TA 1537; (ii) 50 *μ*g/ml, *E. coli WP2 uvrA*; without S9: 2-nitrofluorene—2 *μ*g/ml, TA 98; 4-nitroquinoline-N-oxide—0.1 *μ*g/ml, TA 100; N^4^-aminocytidine—100 *μ*g/ml, TA 1535; 9-amino-1,2,3,4-tetra hydro acridine hydrochloride—15 *μ*g/ml, TA 1537; 4-nitroquinoline-N-oxide—1.0 *μ*g/ml, *E. coli WP2 uvrA*.

**Table 6:** STODI, mutagenicity test results.

Strain	S9	Untreated control	Vehicle control (DMSO)	STODI					Positive control
				78.1 *μ*g/ml	156.2 *μ*g/ml	312.5 *μ*g/ml	625 *μ*g/ml	1250 *μ*g/ml	
*S. typhimurium*									
TA 98	−S9	0.67 ± 1.15	0.33 ± 0.58	2.00 ± 2.00	0.67 ± 1.15	1.00 ± 0.00	0.00 ± 0.00	2.33 ± 0.58	48.00^*∗*^ ± 0.00
	+S9	2.67 ± 2.08	0.67 ± 0.58	1.33 ± 0.58	0.33 ± 0.58	1.33 ± 1.53	1.00 ± 1.00	1.00 ± 1.00	41.00^*∗*^ ± 1.73
TA 100	−S9	2.00 ± 1.00	2.33 ± 0.58	1.67 ± 0.58	1.67 ± 0.58	2.33 ± 1.53	2.33 ± 2.08	0.33 ± 0.58	46.67^*∗*^ ± 0.58
	+S9	4.33 ± 1.53	3.00 ± 0.00	3.67 ± 2.08	4.33 ± 4.04	5.33 ± 2.31	2.67 ± 0.58	6.00 ± 2.00	48.00^*∗*^ ± 0.00
TA 1535	−S9	2.00 ± 1.73	0.33 ± 0.58	0.33 ± 0.58	0.33 ± 0.58	2.67 ± 1.53	0.33 ± 0.58	1.67 ± 0.58	48.00^*∗*^ ± 0.00
	+S9	1.00 ± 1.00	1.00 ± 1.00	1.33 ± 0.58	0.67 ± 1.15	1.67 ± 1.53	1.33 ± 1.15	1.00 ± 0.00	38.33^*∗*^ ± 2.08
TA 1537	−S9	0.00 ± 0.00	0.67 ± 1.15	0.67 ± 1.15	0.33 ± 0.58	1.00 ± 1.00	0.33 ± 0.58	0.33 ± 0.58	40.33^*∗*^ ± 1.53
	+S9	0.67 ± 0.58	0.67 ± 0.58	2.33 ± 0.58	0.33 ± 0.58	1.67 ± 1.53	0.67 ± 0.58	1.67 ± 0.58	42.67^*∗*^ ± 3.21
*E. coli*									
*WP2 uvrA*	−S9	0.33 ± 0.58	0.00 ± 0.00	0.67 ± 0.58	0.00 ± 0.00	0.00 ± 0.00	0.00 ± 0.00	0.00 ± 0.00	39.33^*∗*^ ± 1.53
	+S9	0.67 ± 1.15	0.33 ± 0.58	0.67 ± 1.15	0.00 ± 0.00	0.00 ± 0.00	0.00 ± 0.00	0.00 ± 0.00	34.33^*∗*^ ± 4.04

Revertant colonies *n* = 3 (mean ± SD). ^*∗*^Statistically significant (*P* < 0.05); positive controls. With S9: 2-aminoanthracene (i) 5 *μ*g/ml, TA 98, TA 100, TA 1535, and TA 1537; (ii) 50 *μ*g/ml, *E. coli WP2 uvrA*; without S9: 2-nitrofluorene—2 *μ*g/ml, TA 98; 4-nitroquinoline-N-oxide—0.1 *μ*g/ml, TA 100; N^4^-aminocytidine—100 *μ*g/ml, TA 1535; 9-amino-1,2,3,4-tetra hydro acridine hydrochloride—15 *μ*g/ml, TA 1537; 4-nitroquinoline-N-oxide—1.0 *μ*g/ml, *E. coli WP2 uvrA*.

**Table 7:** Acute oral toxicity test results: clinical signs and gross pathology findings in rats after treatment with PFS.

Test substance	Study	Cage side observations		Period of signs in days, from–to	Gross pathology findings
		Dose (g/kg body weight)	Observed signs		
EGMAX	Sighting (*n* = 1)	2	Nil	0–14	NAD
		5	Nil	0–14	NAD
	Main (*n* = 4)	5	Nil	0–14	NAD
FEED-X	Sighting (*n* = 1)	5	Nil	0–14	NAD
	Main (*n* = 4)	5	Nil	0–14	NAD
KOLIN PLUS	Sighting (*n* = 1)	5	Nil	0–14	NAD
	Main (*n* = 4)	5	Nil	0–14	NAD
PHYTOCEE	Sighting (*n* = 1)	2	Nil	0–14	NAD
		5	Nil	0–14	NAD
	Main (*n* = 4)	5	Nil	0–14	NAD
STODI	Sighting (*n* = 1)	5	Nil	0–14	NAD
	Main (*n* = 4)	5	Nil	0–14	NAD

NAD: no abnormality detected.

**Table 8:** Acute oral toxicity test results: effect of PFS on body weight and percent body weight gain.

Test substance	Study	Dose (g/kg body weight)	Body weight			Percent body weight gain		
			Day 0	Day 7	Day 14	Days 0–7	Days 7–14	Days 0–14
EGMAX	Sighting (*n* = 1)	2	160	186	201	16.25	8.06	25.63
		5	162	192	217	18.52	13.02	33.95
	Main (*n* = 4)	5	164	196.75	206.5	19.97	4.96	25.91
FEED-X	Sighting (*n* = 1)	5	184	220	240	19.57	9.09	30.43
	Main (*n* = 4)	5	181.25	217.25	233.75	19.87	7.61	28.97
KOLIN PLUS	Sighting (*n* = 1)	5	182	214	238	17.58	11.21	30.77
	Main (*n* = 4)	5	181.25	213.25	234.75	17.66	10.09	29.52
PHYTOCEE	Sighting (*n* = 1)	2	170	196	210	15.29	7.14	23.53
		5	172	180	200	4.65	11.11	16.28
	Main (*n* = 4)	5	172	188.75	210	9.74	11.26	22.09
STODI	Sighting (*n* = 1)	5	170	208	236	22.35	13.46	38.82
	Main (*n* = 4)	5	170.5	207.5	237	21.70	14.23	39.01

## Data Availability

All data generated during this study are included in this manuscript and available to access.
